# Long-Term Consumption of Hyaluronan Increases Its Endogenous Levels Correlating with Attenuated Acute Alcohol-Induced Liver Injury

**DOI:** 10.3390/ijms27093941

**Published:** 2026-04-28

**Authors:** Qingkai Zeng, Ziwei Zheng, Ting Sun, Jie Wang, Junqiang Fang, Huarong Shao, Fei Liu, Peixue Ling

**Affiliations:** 1 National Glycoengineering Research Center, Shandong University, Qingdao 266237, China; zengqingkai@mail.sdu.edu.cn (Q.Z.);; 2 Shandong Academy of Pharmaceutical Sciences, Jinan 250101, Chinalfshwu@163.com (F.L.); 3 School of Pharmaceutical Science, Shandong University, Jinan 250012, China; 4 College of Pharmacy, Shandong University of Traditional Chinese Medicine, Jinan 250000, China; 5 Engineering Research Center for Sugar and Sugar Complex, National-Local Joint Engineering Laboratory of Polysaccharide Drugs, Key Laboratory of Carbohydrate and Glycoconjugate Drugs, Shandong Academy of Pharmaceutical Science, Jinan 250101, China

**Keywords:** hyaluronan, acute alcohol-induced liver injury, antioxidant, gut microbiota, liver protection

## Abstract

Inflammation and oxidative stress play important roles in alcohol-induced liver injury. Hyaluronan (HA), a naturally occurring polysaccharide proven to exhibit antioxidant and anti-inflammatory functions, has garnered growing research attention in the field of food in recent years. This study demonstrates that long-term oral administration of HA exerts a protective effect against acute alcohol-induced liver injury (AALI). The findings showed that oral administration of 30, 600, and 1250 kDa HA for 2 and 4 weeks all increased serum and liver HA levels in rats and regulated the composition and abundance of gut microbiota. Meanwhile, oral HA could alleviate the symptoms of liver injury caused by alcohol, including increasing glutathione (GSH) levels, reducing malondialdehyde (MDA) and triglyceride (TG) levels, and decreasing the content of inflammatory factors interleukin-1 beta (IL-1β) and tumor necrosis factor-alpha (TNF-α) compared with the AALI model mice. Furthermore, HA could inhibit the increase in reactive oxygen species (ROS) levels in AML12 cells induced by alcohol and improve the survival rate of alcohol-damaged AML12 cells. In conclusion, this study found that oral administration of HA could increase serum and liver HA levels and has a protective effect on AALI, suggesting the application of HA in health foods for hangover relief and liver protection.

## 1. Introduction

Alcoholic liver disease (ALD), a liver disorder caused by acute or chronic alcohol consumption, is the second most common liver disease, only after viral hepatitis [1,2]. ALD primarily includes fatty liver, hepatitis, hepatic fibrosis, and cirrhosis [3,4]. Among them, fatty liver represents the earliest stage of ALD, characterized by fat deposition in hepatocytes [5]. If left untreated, fatty liver may progress to steatohepatitis, hepatic fibrosis, cirrhosis, and even liver cancer [6,7].

Acute alcohol-induced liver injury (AALI), an ALD caused by acute alcohol consumption, is associated with hepatic oxidative stress and abnormal lipid metabolism [8,9]. After a single short-term intake of a large amount of alcohol, alcohol entering the liver impairs the tricarboxylic acid cycle and inhibits fatty acid metabolism, leading to abnormal lipid accumulation in the liver [10,11]. Meanwhile, alcohol is metabolized to acetaldehyde in the liver via alcohol dehydrogenase (ADH) and the microsomal ethanol oxidizing system, which induces massive production of reactive oxygen species (ROS) [12,13]. This process consumes intracellular reduced glutathione (GSH), causes cellular oxidative stress, promotes intracellular lipid peroxidation to generate a large amount of malondialdehyde (MDA), and triggers endoplasmic reticulum stress and inflammatory responses [14,15,16]. If left untreated after the occurrence of AALI, it may progress to more severe alcoholic liver diseases such as fatty liver and hepatic fibrosis, and even lead to liver cancer [17]. Therefore, thorough exploration of the pathogenesis of AALI and the search for methods to prevent or alleviate AALI are of great significance.

**Figure 1:**
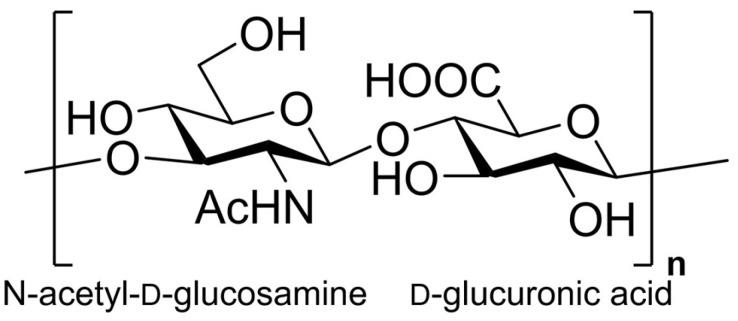
HA chemical structure.

Hyaluronan (HA), a polysaccharide composed of alternating disaccharide units of N-acetyl-D-glucosamine and D-glucuronic acid (Figure 1), is widely present in living organisms [18]. The molecular structure of HA contains a large number of hydroxyl (-OH) and carboxyl (-COOH) groups, which endow HA with excellent hygroscopic and moisturizing properties. Meanwhile, HA exerts antioxidant effects by scavenging free radicals, chelating iron ions, and eliminating ROS via its carboxyl and hydroxyl groups [19,20]. Furthermore, HA also exerts biological activities such as immune regulation and inflammation modulation through binding to HA receptors, including CD44 [21,22].

HA is of significant importance to the liver. During liver injury, the metabolism of HA in the liver becomes abnormal, leading to an increase in HA content in both the liver and serum [23]. In alcoholic liver disease, serum HA concentration has been used as a marker for hepatic fibrosis [24]. In the occurrence of ALD, alcohol intake leads to hepatocyte injury, inflammation, hepatic stellate cell (HSC) activation, and fibrosis, thereby increasing HA synthesis [25,26]. However, the abnormal excessive synthesis of hepatic HA is a consequence of liver injury, rather than a cause of liver damage. Studies have shown that native HA itself does not induce inflammation or damage hepatocytes [26]. It may exert a protective effect against liver injury. Treatment with HA could enhance the proliferation, adhesion, tubule bud formation, and migration abilities of hepatic endothelial cells (ECs), and promote the expression of angiogenic factors VEGF-A and VEGFR1 in hepatic vascular endothelial cells [27]. Damaged liver sinusoidal endothelial cells (LSECs) in liver injury areas could promote injury repair through LSECs that expand and express lymphatic endothelial HA receptor-1 (LYVE-1) at the injury site [28]. This suggests that HA not only does not damage hepatocytes but may also promote the repair of hepatocyte injury.

In recent years, studies have found that oral administration of HA can exert systemic effects. For example, oral HA can increase the content of HA in the skin and joint cavity, thereby enhancing skin hydration and alleviating knee joint pain [29,30], suggesting that oral HA may increase its systemic content. Therefore, we hypothesized that oral administration of HA may also increase its content and exert antioxidant and anti-inflammatory effects in the liver and play a role in preventing and protecting against AALI. Meanwhile, the biological activity of hyaluronic acid is closely related to its molecular weight. The effects of HA with different molecular weights vary significantly, and the molecular weight of orally administered HA may greatly influence its protective efficacy against liver injury. Therefore, this study explored the absorption of HA with different molecular weights in vivo after oral administration and investigated the protective effect of HA on AALI.

## 2. Results

### 2.1. Oral Administration of HA Improves HA Levels In Vivo

**Figure 2:**
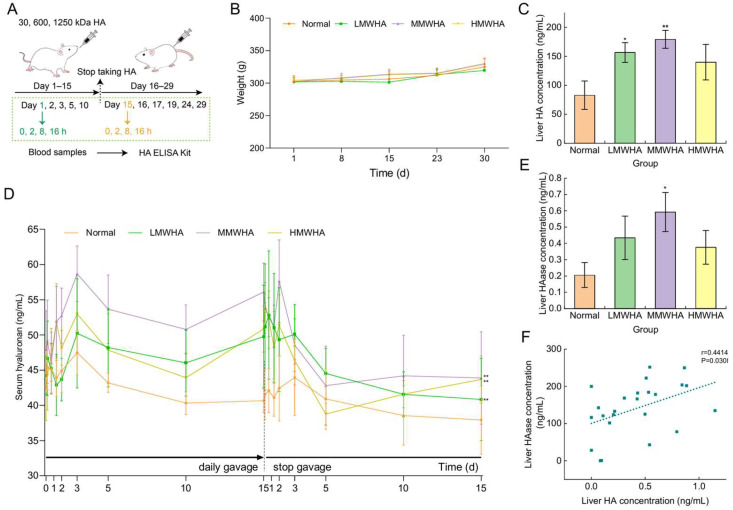
Effects of oral administration of HA in vivo. (**A**) Schematic of the experimental protocol for oral administration of HA. (**B**) Body weight. (**C**) Liver HA levels. (**D**) Serum HA levels. (**E**) Liver HAase levels. (**F**) Correlation analysis. * *p* < 0.05, ** *p* < 0.01 compared with the normal group.

First, the effect of 15-day oral administration of HA on the HA levels in the serum of rats was investigated (Figure 2A). The body weights of the rats in each group were basically the same during the experiment (Figure 2B), indicating that oral administration of HA and blood collection did not affect the health of the rats.

Oral administration of HA increased the HA levels in rat serum, which decreased after the cessation of oral administration (Figure 2D). Among the LMWHA, MMWHA, and HMWHA groups, rats administered MMWHA had the highest HA level in serum. Moreover, the HA content in the serum of rats in each group did not increase indefinitely with the prolongation of oral administration. It only increased rapidly in the first three days, and then remained stable. After stopping oral administration of HA, the serum HA concentration decreased rapidly within the first five days and then stabilized at a level slightly higher than that before oral administration.

Further detection of HA and HAase contents in the liver of mice after 28-day oral administration of HA showed that the contents of HA and HAase in the liver of mice in all HA-administered groups were increased compared with the normal group, with the highest contents observed in the medium-molecular-weight group (Figure 2C,E), which was consistent with the trend of serum HA levels. Meanwhile, the results of correlation analysis indicated a positive correlation between hepatic HA and HAase levels (Figure 2F).

### 2.2. Evaluation of the Safety of 28-Day Oral Administration of HA

**Figure 3:**
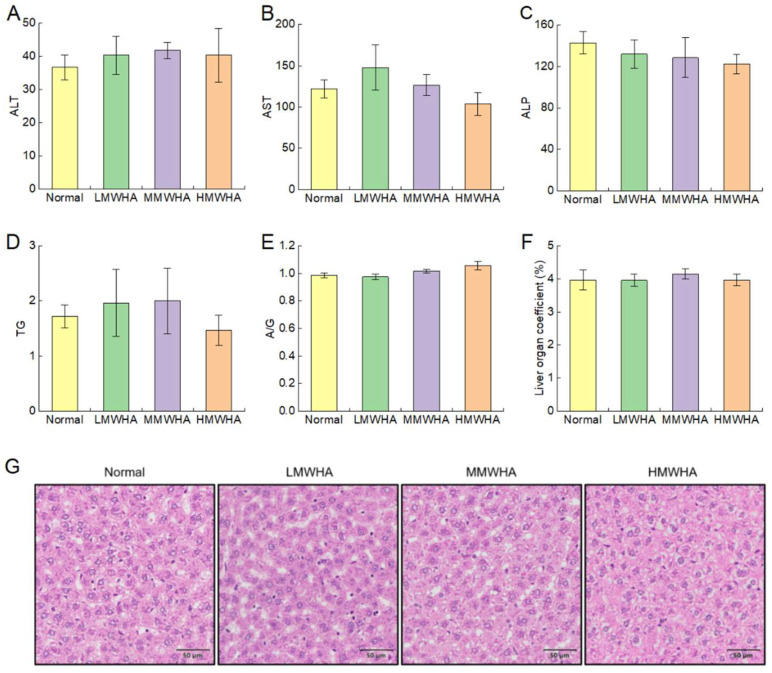
Effects of HA on liver function and tissue. (**A**–**E**) Blood biochemical indices ALT, AST, ALP, TG, and A/G. (**F**) Organ coefficient of the liver. (**G**) H&E staining of the liver.

Serum biochemical (Figure 3A–E) profiling further demonstrated that HA administration did not alter hepatic function markers, including ALT, AST, ALP, and A/G ratios, compared to controls (*p* > 0.05). Quantitative analysis of the organ coefficient of the liver (Figure 3F) showed no statistically significant variations among groups (*p* > 0.05), aligning with H&E findings and indicating neither pathological hypertrophy nor atrophy.

Histopathological evaluation via hepatic H&E staining revealed preserved lobular architecture across all HA-treated groups, comparable to normal controls (Figure 3G). Hepatocytes exhibited intact morphology with centrally located nuclei, devoid of inflammatory infiltration, steatosis, or necrotic foci. The hepatic lobule structure remained intact, with normal distribution of portal triads and bile ducts, and absence of sinusoidal dilatation. The above results indicate that no intergroup differences in histopathological features were observed. The above results indicate that oral administration of HA did not cause liver damage.

**Figure 4:**
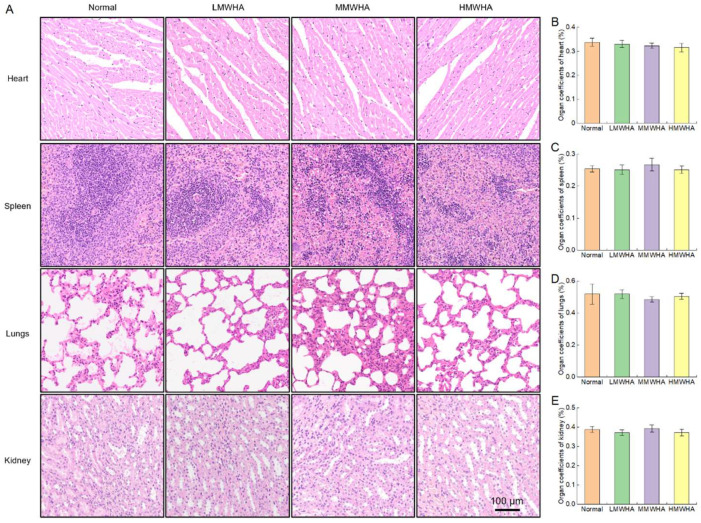
Related organ coefficients and H&E staining (**A**) H&E staining. (**B**–**E**) Organ coefficients.

Furthermore, H&E staining was performed on the heart, liver, spleen, and lung (Figure 4A), and the organ coefficients were calculated by weighing each organ (Figure 4B–E). The H&E staining results showed that in the oral HA groups, the cells of the heart, liver, spleen, and lung tissues of rats had intact morphology and clear tissue structure, with no obvious abnormal pathological changes such as inflammatory infiltration, cell necrosis, edema, or fibrosis, showing no significant difference compared with the normal group. Regarding organ coefficients, the organ coefficients of the heart, liver, spleen, and lung in rats of each group were all within the normal physiological range, and there was no statistical difference between groups (*p* > 0.05). These results indicate that 28-day oral administration of HA did not cause organic damage to the heart, liver, spleen, or lung of rats, exhibiting good safety.

### 2.3. The Impact of HA on the Gut Microbiota in Mice

**Figure 5:**
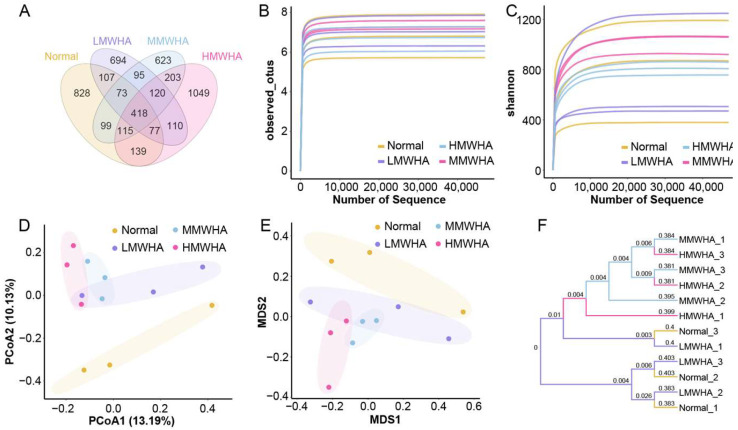
Effect of HA on the gut microbiota in mice. (**A**) The Venn diagram. (**B**,**C**) Multisample rarefaction curves and multisample Shannon–Wiener curves. (**D**) PCoA2. (**E**) NMDS. (**F**) Clustering tree.

Subsequently, the effect of long-term oral administration of HA with different molecular weights on the gut microbiota was investigated. Mice were orally administered 30, 600, and 1250 kDa HA for 28 consecutive days, after which their feces were subjected to 16S rDNA sequencing to analyze the gut microbiota. The Venn diagram (Figure 5A) showed that the Normal, LMWHA, MMWHA, and HMWHA groups shared 418 OTUs, with each group having 828, 694, 623, and 1049 unique OTUs, respectively, suggesting that oral HA changed the gut microbiota composition.

Meanwhile, α-diversity analysis (Figure 5B,C) was used to further evaluate the diversity level of intestinal microbiota in mice from each group. In addition, principal coordinate analysis (PCoA) and nonmetric multidimensional scaling (NMDS) were performed for β-diversity assessment (Figure 5D,E). The PCoA results showed that there were significant differences in the intestinal microbiota community structure between the Normal group and each oral HA group, but the differences among the LMWHA, MMWHA, and HMWHA groups were not significant. Similarly, NMDS showed a large difference between the Normal group and the oral HA groups, as well as similarities among the LMWHA, MMWHA, and HMWHA groups. This also indicates the impact of oral HA on the diversity of intestinal microbiota. Furthermore, Figure 5F shows the similarity among different samples, from which it can be seen that the intestinal microbiota of samples in the Normal group and the LMWHA group were relatively similar, while those in the MMWHA and HMWHA groups were relatively similar.

**Figure 6:**
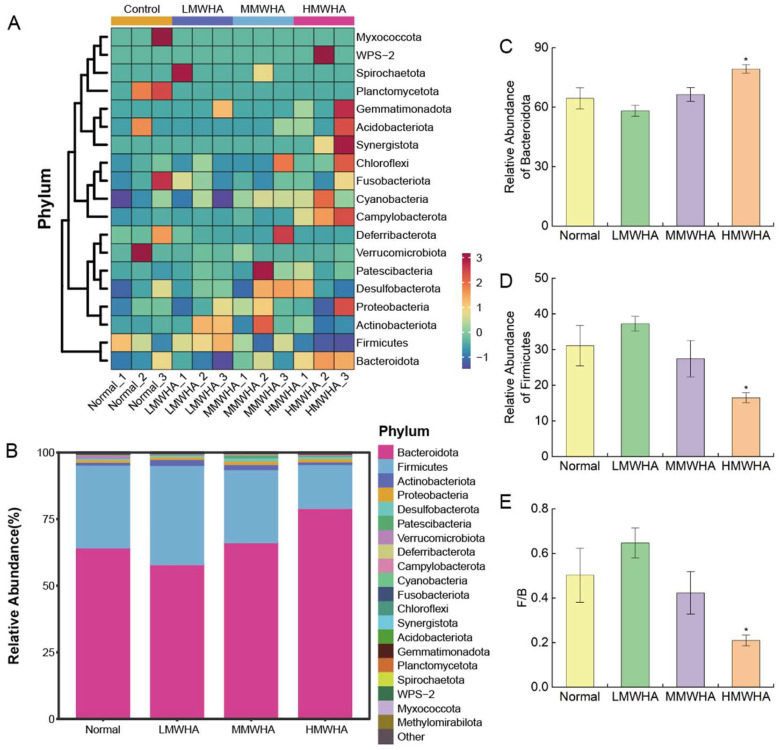
Changes and differences in the gut microbiota. (**A**,**B**) The relative abundance of gut microbiota at the phylum level in the Normal, LMWHA, MMWHA, and HMWHA groups. (**C**,**D**) The relative abundance of Bacteroidetes and Firmicutes. (**E**) The Firmicutes/Bacteroidetes ratio. * *p* < 0.05.

The effect of oral administration of HA with different molecular weights on the abundance of intestinal flora was also evaluated at the phylum level (Figure 6A,B). It can be seen that the abundances of Bacteroidetes and Firmicutes accounted for a relatively high proportion in the mouse intestine, while oral HA altered the relative abundances of these two phyla. High-molecular-weight HA significantly increased the abundance of Bacteroidetes (Figure 6C), decreased the abundance of Firmicutes (Figure 6D), and simultaneously significantly reduced the ratio of Firmicutes-to-Bacteroidetes (Figure 6E).

### 2.4. Long-Term Oral Administration of HA Protects Against AALI

**Figure 7:**
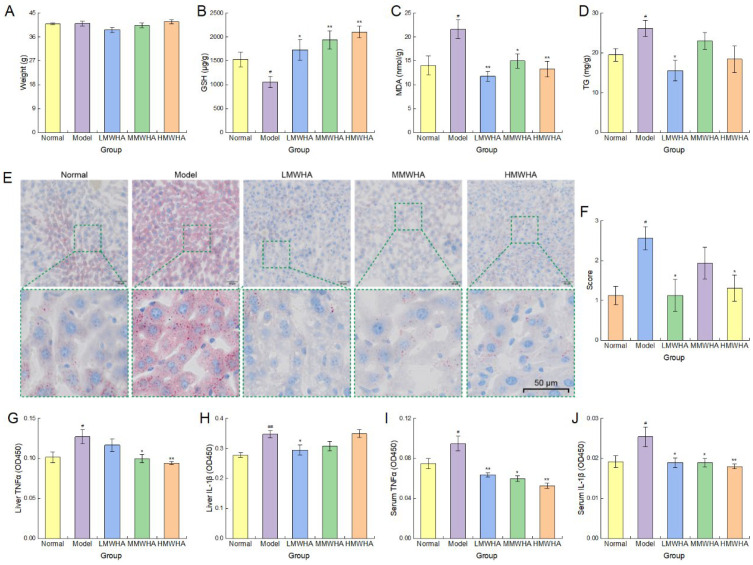
Hepatic protective effect of HA against AALI. (**A**) Weight of mice. (**B**–**D**) Liver GSH, MDA, and TG levels. (**E**,**F**) Liver Oil Red O staining and scoring results. (**G**–**J**) The contents of IL-1β and TNF-α in serum and liver. * *p* < 0.05, ** *p* < 0.01, ^#^ *p* < 0.05, ^##^ *p* < 0.01.

We investigated the protective effect of oral HA on the liver using an AALI model. The body weight of mice in each group remained stable during oral administration of HA (Figure 7A). After mice were given a large amount of alcohol, as shown in Figure 7B–D, compared with the Normal group, the Model group mice had significantly decreased liver GSH content (*p* < 0.05), while MDA and TG contents increased significantly (*p* < 0.05). Meanwhile, the results of liver Oil Red O staining showed that alcohol intake led to abnormal accumulation of lipids in the liver (Figure 7E).

In mice administered oral HA, the liver GSH content in the LMWHA (*p* < 0.05), MMWHA (*p* < 0.01), and HMWHA (*p* < 0.01) groups was significantly higher than that in the Model group, with the enhancing effect being more pronounced as the molecular weight increased (Figure 7B). Meanwhile, the MDA content in the LMWHA (*p* < 0.01), MMWHA (*p* < 0.05), and HMWHA (*p* < 0.01) groups was significantly lower compared to the Model group, and oral administration of LMWHA showed the most obvious effect in reducing MDA (Figure 7C). Moreover, the liver TG content in the LMWHA (*p* < 0.05) group was significantly lower than that in the Model group (Figure 7D). The results of tissue sections also revealed that hepatic lipids in the LMWHA, MMWHA, and HMWHA groups were reduced compared with the Model group (Figure 7E), and the trend of the scoring results (Figure 7F) was consistent with the TG content detected by the kit.

Subsequently, the contents of interleukin-1 beta (IL-1β) and tumor necrosis factor-alpha (TNF-α) in serum and liver were detected. As shown in Figure 7G–J, the contents of IL-1β (*p* < 0.05) and TNF-α (*p* < 0.01) in both serum and liver of mice were elevated in the Model group, indicating that alcohol intake induced hepatic and systemic inflammation [31]. Oral administration of HA, however, inhibited the elevation of the aforementioned inflammatory factors compared with the Model group. The above results indicate that oral administration of HA could improve alcohol-induced abnormalities in hepatic GSH, MDA, and TG, reduce the contents of inflammatory factors TNF-α and IL-1β, alleviate liver inflammation, and thereby exert a liver protective effect.

### 2.5. Protective Effect of HA on Alcohol-Damaged AML12 Cells

**Figure 8:**
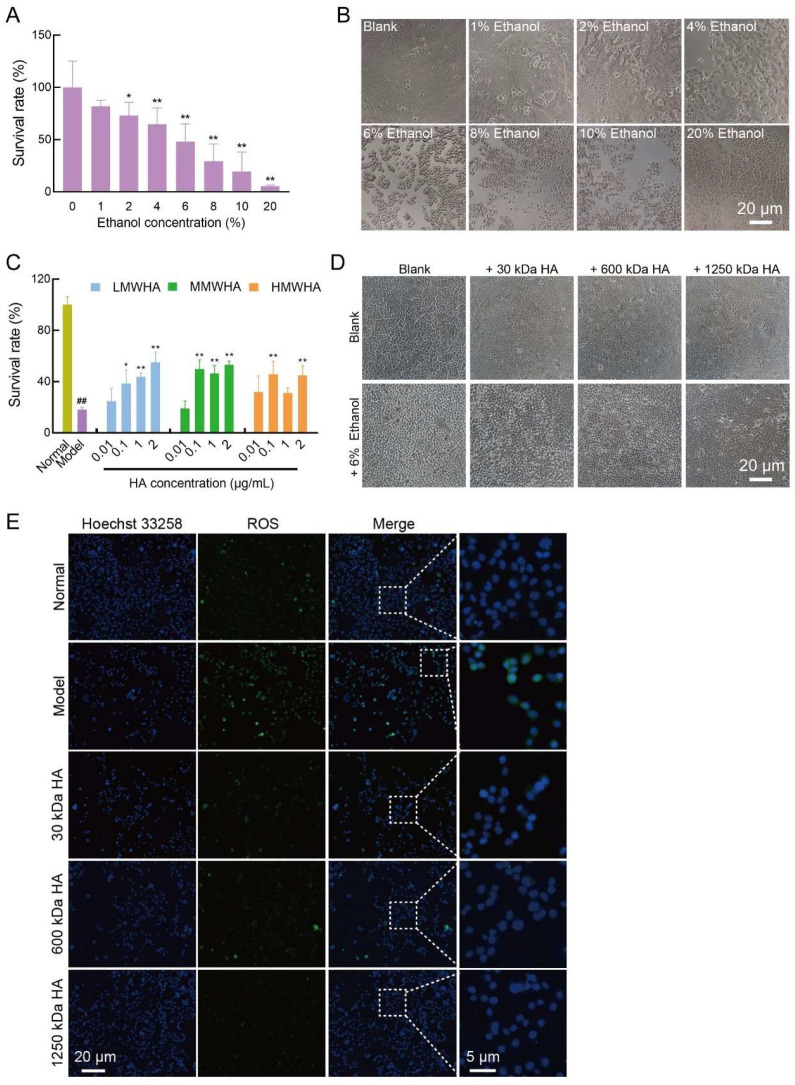
Protective effects of HA on alcohol-damaged AML12 cells. (**A**) Cell viability and (**B**) inverted microscope observation after treatment with different concentrations of alcohol. (**C**) Cell viability and (**D**) inverted microscope observation after treatment with HA. (**E**) ROS staining. * *p* < 0.05, ** *p* < 0.01, ^##^ *p* < 0.01.

Subsequently, the liver protective activity of HA was explored in vitro. First, cells were treated with different concentrations of ethanol. Based on the CCK-8 results and microscopic observations (Figure 8A,B), when the ethanol concentration was 6%, the cell survival rate was approximately 50%, and the cells appeared rounded under the microscope with severe damage. Therefore, 6% ethanol was selected as the concentration for subsequent modeling.

Subsequently, cells were treated with 6% ethanol followed by HA of different molecular weights. The results showed that when the concentration of HA was higher than 0.1 mg/mL, the cell survival rate began to increase significantly compared with the Model group (Figure 8C,D), indicating that HA started to exhibit cytoprotective activity. Through microscopic observation, it was found that after 6% alcohol modeling, the cell edge gap increased and the number of cells decreased, while after adding HA with different molecular weights to the treatment, the state of the cells did not deteriorate significantly after alcohol treatment, which reflected the protective effect of HA on the cells.

We further performed ROS staining on the cells (Figure 8E). The results showed that after alcohol treatment, the intracellular green fluorescence was enhanced, indicating an increase in intracellular ROS. In contrast, the intracellular green fluorescence was reduced in cells pre-incubated with HA followed by alcohol treatment.

## 3. Discussion

Our study is the first to verify the protective effects of long-term oral administration of HA with different molecular weights against acute alcoholic liver injury. Using both animal and cellular experiments, we demonstrated that long-term oral HA administration increased endogenous HA levels, enhanced gut microbiota richness, alleviated alcohol-induced hepatic oxidative stress and ROS production in hepatocytes, and thereby exerted hepatoprotective effects. This study provides a theoretical basis for the development of oral HA dietary supplements and HA-incorporated products for alleviating alcohol-induced liver damage.

Our results revealed that long-term oral administration of HA elevated serum HA levels, indicating that HA of different molecular weights can be absorbed into the body after oral intake. As a macromolecular polysaccharide, HA has been traditionally regarded as being difficult to be directly absorbed through the gastrointestinal tract. With regard to the absorption mechanism of orally administered HA, some studies have proposed that high-molecular-weight HA is degraded into small molecules by the gut microbiota and then absorbed [32,33]. Accordingly, the smaller the molecular weight of orally administered HA, the faster it should be absorbed and the higher its concentration in serum should be. However, our results showed that the serum HA content was the highest following oral administration of medium-molecular-weight HA. A recent study by Liao et al. [34] demonstrated that macromolecular polysaccharides can be directly absorbed into the body via the intestine through the clathrin/dynamin 1/Rab5 pathway, suggesting that a portion of orally administered HA may be directly absorbed through the gastrointestinal tract in its original molecular weight form.

Additional evidence supporting the direct absorption of orally administered HA is that HA of different molecular weights exerts distinct effects following oral intake. Mariko et al. [35] reported that oral administration of 300 kDa HA alleviated wrinkles more rapidly than 2 kDa HA. In a photoaging model, Chinatsu et al. [36] found that 10 kDa HA reduced skin thickening, whereas 300 kDa HA mitigated the decrease in skin hydration. Our study also demonstrated that in AALI, although MMWHA resulted in higher hepatic HA content after oral administration, HMWHA better alleviated the symptoms of AALI. If HA were completely degraded in the intestine before being absorbed into the body, orally administered HA of any molecular weight would produce identical effects. Therefore, at least a portion of orally administered HA must be directly absorbed into the body without degradation, which further corroborates the finding by Liao et al. [34] that macromolecular polysaccharides can be directly absorbed through the intestine via the clathrin/dynamin 1/Rab5 pathway.

The most significant increase in serum HA levels was observed after oral administration of MMWHA. This may be attributed to the fact that both LMWHA and MMWHA are absorbed more rapidly than HMWHA. However, LMWHA that enters the bloodstream is degraded faster than MMWHA. At the time of detection, the absorbed LMWHA had been degraded into HA oligosaccharides, which are not detectable by the assay kit. Thus, the highest systemic HA levels were presented in the MMWHA group.

Oral HA administration also upregulated the expression of hepatic HAase, which may be linked to the initial increase followed by a subsequent decrease in serum HA levels during oral HA supplementation. The liver is an important site for HA metabolism in the body, where hepatic stellate cells can synthesize HA, and liver sinusoidal endothelial cells are the primary sites for HA degradation in the circulatory system [37]. Orally administered HA first passes through the gastrointestinal tract and then enters the liver via the bloodstream [38]. Hyal-1 activity can be detected in the lysosomes of mouse liver, indicating that the liver is capable of degrading high-molecular-weight HA [39]. Therefore, the trend whereby serum HA content first increased and then stabilized may be attributed to the elevated hepatic HAase expression induced by oral HA. Oral HA upregulated the expression of HAase in the liver, accelerating the rate of HA degradation and thereby establishing a new equilibrium between HA intake and catabolism. Following the cessation of oral HA administration, serum HA levels declined to the pre-administration baseline, suggesting that continuous oral HA supplementation is necessary to maintain relatively high HA levels in vivo. However, whether the upregulation of HAase expression is indeed induced by oral HA administration, as well as the specific mechanism underlying the upregulation of hepatic HAase expression induced by oral HA remains unclear and requires further research for verification.

As serum HA level serves as a marker for hepatic fibrosis [40,41], the increase in HA content in both the liver and serum after oral administration of HA may be a result of liver injury. Therefore, we evaluated the safety of 28-day oral administration of HA in rats. Safety assessments confirmed that the elevated HA levels in both serum and liver after oral HA administration were attributable to the absorption of HA into the body, rather than to liver injury.

Long-term oral administration of hyaluronic acid (HA) of three distinct molecular weights also enhanced the richness of the gut microbiota. Studies have shown that oral administration of HA oligosaccharides (o-HAs) can regulate the gut microbiota and improve its abundance [42]. The above results further reveal that HA with low, medium, and high molecular weights also exerts similar effects. Notably, the mechanism by which HA influences gut microbiota remains unclear. Studies have indicated that oral HA promotes the growth of *Bacteroides*, *Parabacteroides*, and *Faecalibacterium*, likely due to the higher utilization efficiency of these bacteria for HA [43,44]. Additionally, HA may alter the viscosity of the chyme or intestinal transit time, thereby exerting an indirect effect on the gut microbiota.

Similarly, our study demonstrated that HMWHA significantly elevated the relative abundance of Bacteroidetes, reduced that of Firmicutes, and markedly decreased the Firmicutes/Bacteroidetes ratio. Notably, Bacteroidetes are beneficial for carbohydrate metabolism and intestinal barrier function, so the increased abundance induced by HMWHA may help strengthen gut health. Meanwhile, the Firmicutes/Bacteroidetes ratio is considered a key indicator of gut microbiota health and is commonly associated with diseases such as obesity [45,46], and its reduction by HMWHA suggests a regulatory role in maintaining intestinal homeostasis. These findings indicate that HA’s regulation of gut microbiota is related to its molecular weight, which may contribute to its liver-protective effect.

Elevated hepatic HA levels were observed following oral HA administration. Given the antioxidant and anti-inflammatory properties of HA, it may thereby exert a hepatoprotective effect. The hepatoprotective activity of orally administered HA was verified in mice with AALI. After ethanol gavage, hepatic GSH levels were decreased, while hepatic MDA and TG levels were increased in mice. In addition, the levels of the pro-inflammatory cytokines TNF-α and IL-1β were elevated in both serum and liver tissue. Alcohol entering the liver impairs the tricarboxylic acid cycle and inhibits fatty acid metabolism, leading to increased hepatic TG and abnormal lipid accumulation [47,48]. Meanwhile, a large amount of ROS generated during alcohol metabolism consumes intracellular GSH and promotes intracellular lipid peroxidation, resulting in the production of a large quantity of MDA [49,50]. Therefore, the above results indicate that the mouse model of AALI was successfully induced.

In mice receiving oral HA administration, elevated GSH levels were observed, and the higher the molecular weight of the orally administered HA, the greater the increase in GSH content. Conversely, LMWHA induced a more pronounced reduction in MDA and TG levels. Collectively, these findings suggest that LMWHA, similar to HMWHA, provides superior protection against liver injury compared to MMWHA, despite the latter resulting in higher serum HA levels following oral intake. Meanwhile, the results for TNF-α and IL-1β indicated that HMWHA exhibited a better efficacy in mitigating hepatic and systemic inflammation. This finding is also consistent with previous literature reporting that orally administered HMWHA alleviates systemic inflammation more effectively than MMWHA and LMWHA [51].

In cellular experiments using an alcohol-injured mouse hepatic AML12 cell model, HA also elevated cell viability and reduced intracellular ROS levels. A large amount of ROS is generated during intracellular alcohol metabolism, which may induce cellular oxidative stress and subsequently lead to cell damage [31]. Our results suggest that HA can decrease intracellular ROS levels, alleviate oxidative stress, and thereby protect cells from alcohol-induced injury.

In mouse experiments, alcohol-induced alterations in hepatic indicators such as reduced glutathione, together with elevated levels of inflammatory factors including IL-1β and TNF-α, reflected aggravated liver injury and inflammation [52,53]. The in vitro cellular experimental results indicate that the hepatoprotective effect of HA may be achieved by alleviating oxidative stress.

However, this cannot explain the complex effects of HA with different molecular weights on hepatic MDA, TG, GSH, TNF-α, and IL-1β levels. Therefore, the pathways through which HA exerts its hepatoprotective effects may not be singular. The results of gut microbiota experiments suggest that HA may also exert a protective effect against liver injury by regulating the gut microbiota. For example, studies have shown that an increase in *Bacteroides acidifaciens* in the intestine alleviates alcohol-induced liver injury [54]. Additionally, HA may also act on HA receptors such as CD44 and TLR4 on the intestinal tract before absorption, exerting an anti-inflammatory effect by protecting the gastrointestinal tract or via the gut/brain/liver axis [55,56].

In the field of liver protection, Silymarin and Bicyclol have been proven to exert a hepatoprotective effect [57,58,59]; however, the mechanisms by which HA exerts its effects are distinctly different from those of these agents. Both silymarin and bicyclol can rapidly ameliorate inflammation and oxidative stress following oral administration, whereas HA exerts a preventive effect against AALI by slowly increasing hepatic HA content and improving the gut microbiota through long-term oral supplementation. Meanwhile, as a substance that is naturally abundant in the human body, long-term oral HA supplementation poses no safety concerns. Therefore, compared with other agents for the treatment of liver injury, HA is more suitable for use as a health supplement or a daily food additive. Its role is not to treat liver injury after it has occurred, but to prevent it before onset. However, this study only investigated the effects of HA with different molecular weights, and did not study the dose-effect of HA, which needs further in-depth research.

## 4. Materials and Methods

### 4.1. Materials

The 30 kDa and 600 kDa HA were provided by the Shandong Academy of Pharmaceutical Sciences (Jinan, China), while the 1250 kDa HA (cosmetic grade) was purchased from Huaxi Bio-Tech Co., Ltd. (Jinan, China). The triglyceride (TG) assay kit, malondialdehyde (MDA) assay kit, and reduced glutathione (GSH) assay kit were obtained from Beijing Solarbio Science & Technology Co., Ltd. (Beijing, China). The ROS assay kit was provided by Beyotime Biotechnology (Shanghai, China). All enzyme-linked immunosorbent assay (ELISA) kits were ordered from the Jiancheng Bioengineering Institute of Nanjing (Nanjing, China). AML12 cells were purchased from BeNa Culture Collection (Xinyang, China).

Preparation of HA solutions: 5 mg/mL LMWHA (30 kDa), MMWHA (600 kDa), and HMWHA (1250 kDa) solutions were prepared by dissolving 50 mg of each HA in 10 mL of ddH_2_O, followed by vortexing and standing overnight at 4 °C for complete dissolution, and stored at 4 °C until use.

### 4.2. Animals

All animals were specific pathogen-free (SPF) grade and obtained from Jinan Pengyue Experimental Animal Breeding Co., Ltd. (Jinan, China). The rats and mice were confined in animal cages and housed at the New Drug Evaluation Center of Shandong Academy of Pharmaceutical Sciences with a regulated temperature (24 ± 1) °C, (55 ± 5)% humidity, 12 h dark/12 h light cycle, with free access to food and water, and were subjected to a one-week adaptive feeding before experimental treatment. The study was approved by the Laboratory Animal Ethics Committee of Shandong Academy of Pharmaceutical Sciences and performed in the Laboratory Animal Center of Shandong Academy of Pharmaceutical Sciences (Permit Number: IACUC-2023-M014).

### 4.3. The Effects of Oral Administration of HA In Vivo

#### 4.3.1. Measurement of Serum HA Levels

SPF grade healthy SD female rats were weighed and randomly divided into four groups (*n* = 6 per group): Normal group, LMWHA group (5 mg/mL 30 kDa HA, 20 mg/kg), MMWHA group (5 mg/mL 600 kDa HA, 20 mg/kg), and HMWHA group (5 mg/mL 1250 kDa HA, 20 mg/kg). The oral dose of 20 mg/kg HA administered to rats was converted to the human equivalent dose (HED) based on body surface area scaling, which corresponds to approximately 200 mg of HA per day for humans. The specific calculation is as follows [60]:

The formula for HED conversion is:

HED (mg/kg) = Animal dose (mg/kg) × (Animal K_m_/Human K_m_)

Rearranged for rat dose:

Rat does (mg/kg) = HED (mg/kg) × (Human K_m_/Rat K_m_)

Assuming a human body weight of 60 kg, the HED of 3.33 mg/kg was calculated based on a daily human intake of 200 mg HA. Specifically, Human Km = 37 and Rat Km = 6, yielding a calculated rat equivalent dose of 20.535 mg/kg. Therefore, rats were administered 20 mg/kg HA daily, which is equivalent to a daily human intake of 200 mg HA. This dose falls within the normal range of oral HA intake reported in the literature for human subjects [61,62].

Rats in the HA-treated groups were gavaged daily with HA solutions, whereas those in the Normal group received ddH_2_O. The entire experimental period lasted 30 days, with daily gavage performed for the first 15 days and no gavage for the subsequent 15 days.

Blood samples were collected from the jugular fossa of rats during the gavage period. On the first day and the last day (day 15) of oral HA administration, blood was collected 4 times daily, specifically before administration and at 2, 8, and 16 h after administration. On days 2, 3, 5, and 10 of oral HA administration, blood was collected once daily, before administration. Additionally, following the termination of gavage on day 15, blood samples were collected on days 2, 3, 5, 10, and 15 post-termination.

The obtained blood samples were allowed to stand for 20 min at 4 °C in a refrigerator, followed by centrifugation in a centrifuge at 8000 rpm at 4 °C for 20 min, and the supernatant was taken and stored at −20 °C. After all serum samples were collected, the HA content in rat serum was detected using the rat HA ELISA kit (CAS 9004-61-9).

#### 4.3.2. Blood Routine and Blood Biochemistry

After the final blood sampling, the rats were anesthetized and then sacrificed. Prior to sacrifice, blood was collected from the abdominal aorta using anticoagulant tubes and coagulation-promoting tubes. Blood from anticoagulant tubes was used for blood routine tests, while blood from coagulation-promoting tubes was centrifuged to obtain serum for serum biochemical evaluations, including liver function indices such as alanine aminotransferase (ALT), aspartate aminotransferase (AST), alkaline phosphatase (ALP), and albumin/globulin (A/G). Serum biochemicals were measured with a biochemical analyzer and performed at the New Drug Evaluation Center of Shandong Academy of Pharmaceutical Sciences.

#### 4.3.3. Organ Coefficient

After blood collection and sacrifice, the rats’ livers, hearts, spleens, lungs, and kidneys were harvested. These organs were rinsed with physiological saline, blotted dry, and weighed. Organ coefficients were calculated using the formula:

Organ coefficient (%) = Organ weight (g)/Body weight (g) × 100%

#### 4.3.4. H&E-Stained Sections

Tissue samples from the liver, heart, spleen, lung, and kidney were each preserved in 4% paraformaldehyde for subsequent H&E staining and sectioning. All sections were prepared by Servicebio Company and scanned using the VS120 whole-slide digital scanning microscope (OLYMPUS, Tokyo, Japan) for histopathological assessment via imaging analysis.

#### 4.3.5. Gut Microbiota Analysis

SPF grade healthy ICR female mice were weighed and randomly divided into four groups: Normal group, LMWHA group (5 mg/mL 30 kDa HA, 30 mg/kg), MMWHA group (5 mg/mL 600 kDa HA, 30 mg/kg), and HMWHA group (5 mg/mL 1250 kDa HA, 30 mg/kg). The gavage dose was calculated according to the method described in Section 4.3.1. Using a Mouse K_m_ = 3, the theoretical equivalent dose for mice was 41.07 mg/kg. Considering that the mice used in this study had a higher body weight (approximately 40 g, with a standard Km = 3 for a 20 g mouse), and the K_m_ factor increases proportional to W^2/3^ within a species as body weight increases [60], the actual gavage dose was adjusted to 30 mg/kg. Mice in the HA-treated groups were gavaged daily with HA solutions, whereas those in the Normal group received an equal volume of ddH_2_O. The fecal samples were collected and flash-frozen in liquid nitrogen for gut microbiota analysis.

#### 4.3.6. Measurement of Liver HA and HAase

On the final day of gavage, the mice were euthanized by cervical dislocation, followed by liver excision. The liver tissues were accurately weighed (approximately 0.1 g) and homogenized in PBS solution (1 mL PBS per 0.1 g liver tissue) using a high-throughput tissue grinder at 60 Hz for 60 s. The homogenates were then sonicated at 4 °C for 20 min using a cell disruptor, followed by centrifugation at 8000 rpm at 4 °C; supernatants were collected for analysis. Liver HA content was measured using a mouse HA ELISA kit, and liver hyaluronidase (HAase) level was assayed with a mouse HAase ELISA kit.

For serum HA detection in mice, the obtained blood samples were allowed to stand for 20 min at 4 °C in a refrigerator, followed by centrifugation in a centrifuge at 8000 rpm at 4 °C for 20 min, and the supernatant was taken and stored at −20 °C. After all serum samples were collected, the HA content in rat serum was detected using the mouse HA ELISA kit.

### 4.4. Protective Effect of HA on Ethanol-Induced Liver Injury Mice Model

#### 4.4.1. Model Establishment and Administration

SPF grade healthy ICR male mice were weighed and randomly divided into five groups (*n* = 8 in each group): Normal group, Model group, LMWHA group (5 mg/mL 30 kDa HA, 30 mg/kg), MMWHA group (5 mg/mL 600 kDa HA, 30 mg/kg), and HMWHA group (5 mg/mL 1250 kDa HA, 30 mg/kg). Mice in the HA treatment groups received oral administration of both HA and ethanol. The model group, serving as the negative control, received oral ethanol only without HA administration. The normal group, as the normal control, received neither ethanol nor HA orally.

Specifically, mice in the HA-treated groups were gavaged daily with HA solutions, whereas those in the Normal group and Model group received ddH_2_O. On the last day of the 28-day administration, mice in the Model group and all HA groups were given a single gavage of 50% ethanol solution at a dose of 12 mL/kg body weight. The modeling method was modified based on the protocol described in previous studies [63,64]. After 16 h of fasting, the mice were euthanized. Liver tissues were harvested, and part of the tissues was snap-frozen in liquid nitrogen and stored at −80 °C for subsequent tests, and the other part was fixed in paraformaldehyde solution for frozen section preparation and oil-red O staining.

#### 4.4.2. Evaluation of TG, MDA, GSH, TNF-α, and IL-1β in Mice Livers

TG, MAD, and GSH content in mouse liver tissues was extracted and detected using the TG, MDA, and GSH assay kit. TNF-α and IL-1β levels were quantified using the mouse TNF-α ELISA kit and IL-1β ELISA kit.

#### 4.4.3. AML12 Cell Alcohol-Injured Model

AML12 cells were digested by trypsin and seeded into 6-well plates at a density of 2 × 10^4^ cells per well, which were then incubated in a 37 °C, 5% CO_2_ incubator. The modeling method was modified based on the protocol described in previous studies [65]. Briefly, when cells grew to approximately 60% confluency, they were treated with ethanol-containing medium at a series of concentrations (1%, 2%, 4%, 6%, 8%, 10%, 20%), with 2 mL of medium per well. After incubation with each concentration of alcohol, cellular morphological changes were observed under a microscope. Cell viability was determined using the CCK-8 assay. Absorbance was measured at 450 nm using a TECAN Infinite M200PRO microplate reader to identify the optimal concentration for subsequent modeling.

#### 4.4.4. Cell Viability

Logarithmically growing AML12 cells were dispersed into single-cell suspensions by pipetting, counted, and seeded into culture plates at a density of 3000 cells per well, which were then incubated in a 37 °C, 5% CO_2_ incubator until cell adhesion.

Under the selected 6% alcohol modeling condition, HA-containing medium of low (30 kDa), medium (600 kDa), and high (1250 kDa) molecular weights were prepared at different concentrations (0.01, 0.1, 1, 2 μg/mL). AML12 cells were first incubated overnight with HA of each concentration and molecular weight, followed by the addition of a mixture containing alcohol and trans HA for 2 h of incubation, with 2 mL of medium per well. At the end of incubation, cell viability was assessed via the CCK-8 method, and cell status was observed under a microscope, including changes in intercellular gaps and cell count.

#### 4.4.5. ROS-Related Detection

Logarithmically growing AML12 cells were dispersed into single-cell suspensions by pipetting, counted, and seeded into culture plates at a volume of 2 mL medium per well, which were then incubated in a 37 °C, 5% CO_2_ incubator until cell adhesion. Subsequent group treatments were performed as follows: the control group was incubated with 2 mL of regular culture medium per well throughout; the alcohol-injured group was first incubated overnight with 2 mL of regular culture medium per well, followed by a 2-h incubation with 2 mL of medium containing 6% alcohol per well; the HA intervention groups were first incubated overnight with 2 mL of regular culture medium per well supplemented with HA of different molecular weights (30, 600, 1250 kDa) and concentrations (0.5, 1, 2 mg/mL), then incubated for 2 h with 2 mL of a mixture of 6% alcohol and the corresponding HA per well. At the end of incubation, cells were rinsed with PBS and loaded with ROS fluorescent probe working solution according to the kit instructions, after which intracellular ROS levels were quantitatively analyzed via fluorescence microscopy.

#### 4.4.6. Statistical Analysis

Data were analyzed by OriginPro 2025 and GraphPad Prism 8.0.1. Data between two groups were analyzed using a *t*-test. Data from more than two groups were determined using one-way analysis of variance (ANOVA) combined with Dunnett’s post-hoc test, and a value of *p* < 0.05 was considered statistically significant. All data are expressed as the mean ± standard error of the mean (SEM), and error bars represent the standard error of the mean.

## 5. Conclusions

In conclusion, oral administration of LMWHA, MMWHA, and HMWHA can effectively enhance serum and liver HA levels with a good safety profile, regulate the composition and abundance of gut microbiota, and has a certain protective effect on AALI. However, the specific action mechanism of HA in vivo has not been completely clarified. This protective effect may be achieved through a combination of the direct antioxidant and anti-inflammatory effects exerted by HA entering the liver and mechanisms such as gut microbiota regulation, and the specific mechanism still requires subsequent studies. The aforementioned effects of long-term oral HA suggest its potential application in hangover-relieving and liver-protecting health supplements and daily liver-protecting food additives.

## Data Availability

The original contributions presented in this study are included in the article. Further inquiries can be directed to the corresponding authors.

## Abbreviations

The following abbreviations are used in this manuscript:

AALI	acute alcohol-induced liver injury
ADH	alcohol dehydrogenase
ALP	alkaline phosphatase
ALT	alanine aminotransferase
AST	aspartate aminotransferase
ALD	alcoholic liver disease
AML12	alpha mouse liver 12
ANOVA	one-way analysis of variance
A/G	albumin/globulin
CCK-8	cell counting kit-8
ECs	endothelial cells
ELISA	enzyme-linked immunosorbent assay
GSH	glutathione
HA	hyaluronan
HAase	hyaluronidase
HMWHA	high molecular weight HA
HSC	hepatic stellate cell
IL-1β	interleukin-1 beta
LSECs	liver sinusoidal endothelial cells
LYVE-1	lymphatic endothelial HA receptor-1
LMWHA	low molecular weight HA
MDA	malondialdehyde
MMWHA	medium molecular weight HA
NMDS	nonmetric multidimensional scaling
OTUs	operational taxonomic units
o-HAs	oligosaccharides
PCoA	principal coordinates analysis
ROS	reactive oxygen species
SPF	specific pathogen free
TG	triglyceride
TNF-α	tumor necrosis factor alpha
VEGF-A	vascular endothelial growth factor A
VEGFR1	vascular endothelial growth factor receptor 1
